# Help to Overcome Problems Effectively for Cancer Survivors: Development and Evaluation of a Digital Self-Management Program

**DOI:** 10.2196/17824

**Published:** 2020-05-19

**Authors:** Faith Martin, Hayley Wright, Louise Moody, Becky Whiteman, Michael McGillion, Wendy Clyne, Gemma Pearce, Andy Turner

**Affiliations:** 1 Faculty of Health and Applied Science University of the West of England Bristol United Kingdom; 2 Faculty Research Centre for Intelligent Healthcare Faculty of Health and Life Sciences Coventry University Coventry United Kingdom; 3 Faculty Research Centre for Arts, Memory and Communities Faculty of Arts and Humanities Coventry University Coventry United Kingdom; 4 UK Early Cancer Detection Consortium Faculty of Health and Life Sciences Coventry University Coventry United Kingdom; 5 Baxter Healthcare Compton Newbury United Kingdom; 6 School of Nursing McMaster University Hamilton, ON Canada; 7 Hope for The Community, Community Interest Company The Enterprise Hub Coventry United Kingdom; 8 National Institute for Health Research, Research Design Service South West Peninsula Medical School Plymouth University Devon United Kingdom; 9 Faculty of Health and Life Sciences School of Psychological, Social and Behavioural Sciences Coventry University Coventry United Kingdom

**Keywords:** positive psychology, self-management, hope, quality of life, survivorship, cancer

## Abstract

**Background:**

People living with cancer face numerous psychosocial challenges, including cancer-related fatigue, fear of recurrence, and depression. There is a lack of digital interventions tailored to the needs of people living with all types of cancer. We developed a 6-week, digital, peer-delivered, self-management program: iHOPE (Help to Overcome Problems Effectively; where ‘i’ indicates the digital version of the program). The program is underpinned by positive psychology and cognitive behavioral therapy to meet these psychosocial challenges.

**Objective:**

This study aimed to assess the feasibility of the iHOPE program among people living with cancer. Program adherence and satisfaction along with changes in psychological distress and positive well-being were measured.

**Methods:**

A pre-post, acceptability, and feasibility design was used. People living with cancer (N=114) were recruited via a national cancer charity in the United Kingdom and were given access to the iHOPE program. Demographic and other participant characteristics were recorded. Participants completed digital measures at baseline and the end of the 6-week program for depression, anxiety, cancer-related fatigue, cancer *worry* or fear of cancer recurrence, positive mental well-being, hope, gratitude, and health status. The website’s system recorded data on the usage of the program. Satisfaction with the program was also measured.

**Results:**

A total of 114 participants completed the baseline questionnaires. Of these, 70 people (61.4%) participated in all 6 sessions. The mean number of sessions undertaken was 5.0 (SD 1.5). Moreover, 44.7% (51/114) of participants completed at least three sessions and end-of-program outcome measures. A total of 59 participants completed the satisfaction questionnaire, where ≥90% (54/58) of participants reported that the program was easy to navigate and was well managed by the peer facilitators, and that they found the social networking tools useful. Preliminary efficacy testing among the 51 participants who completed baseline and postprogram outcome measures showed that postprogram scores decreased for depression, anxiety, cancer-related fatigue, and fear of recurrence (all *P*<.001) and increased for positive mental well-being (*P*<.001), hope (both *P*<.001), and gratitude (*P*=.02).

**Conclusions:**

The feasibility evidence is promising, showing that the peer-delivered digital iHOPE program is acceptable and practical. Implementation of the iHOPE program on a wider scale will incorporate further research and development to maximize the completion rates of the measures. Initial effectiveness data suggest positive impacts on important cancer-related quality of life and mental well-being outcomes. A randomized controlled trial design with a longer follow-up is needed to confirm the potential of the iHOPE program for improving mental and physical health outcomes for cancer survivors.

## Introduction

### Background

Globally, there were an estimated 18.1 million new cases of cancer in 2018 [1]. The worldwide incidence of cancer is predicted to rise by 75% over the next two decades [2], leading to a world cancer burden of around 25 million cases by 2030 [3]. Globally, there are around 43.8 million people living 5 years beyond their diagnosis [1]. On the basis of the most recently collected national datasets in the United Kingdom, 4.7 million new cases of cancer were detected in 2018, with 13.5 million people living 5 years beyond their diagnosis [4]. The number of cancer survivors in the United Kingdom alone is projected to increase by approximately 1 million per decade from 2010 to 2040 [5]. Combined with effective treatments, this leads to a growing population of cancer survivors, many with unmet needs, and experiencing psychosocial and physical difficulties.

Cancer survivors face a number of challenges following primary treatment, including fatigue, pain, sexual problems, cognitive functioning, fear of cancer recurrence, depression, anxiety, social isolation, and financial issues [6-8]. A substantial number of studies report such difficulties in the long term, particularly when treatment ends and contact with health care professionals diminishes, patients often report feeling abandoned, vulnerable, and as if they have lost the *safety net* they felt they had during treatment [7]. Clinically significant cancer-related fatigue is common [9,10]. Research suggests that after treatment has ended, a significant proportion of cancer patients experience fear of recurrence [11], with potential long-term negative impacts on quality of life and mental health, including hypervigilance, anxiety, posttraumatic stress, and depression [11-16]. Support for fear of cancer recurrence is an unmet need for between 22% and 90% of cancer survivors [12]. Overall, 12% to 20% of survivors of cancer meet diagnostic criteria for major depression and 18% to 40% for an anxiety disorder in the first 2 years following diagnosis [15,16]. Although these problems are frequently encountered in clinical practice, no clear consensus exists on the best management strategies to support people experiencing anxiety and depression posttreatment.

In the United Kingdom, the National Health Service (NHS) national health care strategy has identified the important role that technology can play in supporting patients with long-term conditions to be better able to self-manage their health [17,18]. This builds on the UK National Cancer Survivorship Initiative, which highlighted the need for a greater focus on recovery, health, and well-being after cancer treatment [19]. Digital self-management interventions are then a central feature of future plans to support cancer survivors.

Self-management interventions have been found to improve outcomes, including quality of life and health care utilization in long-term conditions [20]. Although there are substantial issues concerning intervention fidelity and content, implementation, and scaling and reach of intervention delivery, findings from a review and meta-analysis of digital self-management interventions for cancer survivors found generally positive, support for this approach [21]. A recent systematic review of all cancer self-management interventions highlighted the enormous diversity in intervention content, rendering it difficult to draw conclusions about their effectiveness, and found that there was very poor sustainability of interventions [22]. Furthermore, it is vital to note that many of these research-based interventions are not openly available to the public, with poor implementation observed [23]; indeed, there are very few self-management programs actually available in the United Kingdom [24]. Partnering with implementation stakeholders at an early stage in intervention development is advised to address this challenge [25].

In response to the shortage of available, tailored self-management support programs for cancer survivors, we worked with cancer survivors, clinicians, and other experts to develop a group-based, face-to-face self-management program: Help to Overcome Problems Effectively, known as the *HOPE* program, for survivors of all types of cancer. A recent systematic review of self-management interventions showed that content was largely based on expert opinions or previous models of self‐management or chronic care, with patient input into the design reported in only about 10% of the studies [26]. Therefore, the involvement of cancer survivors in the development, testing, and facilitation of the HOPE program is a particular strength of the intervention compared with other cancer self-management programs. Furthermore, the HOPE program is novel and distinct from many other cancer self-management programs because of its roots in positive psychology [27-29] and its unique focus on hope and gratitude to create an upward spiral of positivity [30] to improve well-being and coping. Fredrickson [31] shows that increasing positive emotions broadens attention, thinking, and action, which enables people to develop more creative thought and action pathways (eg, expanding coping skills), and thus develop crucial personal and social resources for self-management.

Hope theory [32,33] is similar to self-efficacy theory, but the latter focuses on specific goals and behaviors, whereas hope theory recognizes enduring cross-situational goals and behaviors, and as such, hope theory is better suited to the complexity of managing the diverse impact of long-term conditions. Goals are fundamental in hope theory, which encompasses a cognitive set that is based on both agency (goal-directed determination) and pathways (planning ways of achieving goals).

Gratitude has been shown to improve psychological well-being and increase positive emotions [34,35], with some interventions showing that increasing gratitude is linked to improvements in depression [36]. A gratitude activity is a weekly feature in the HOPE program and is designed to increase participants’ positive emotions. The HOPE program also includes other evidence-based cognitive behavioral therapy and positive psychological activities such as identifying personal strengths, scheduling pleasant activities, mindfulness, relaxation training, and reviewing successes.

Group curative factors of instilling hope, universality, and altruism [37] are embedded within the HOPE program content, where participants observe their peers overcoming challenges and achieving goals (*instillation of hope*), share experiences (*universality*), and provide informational and emotional support for each other (*altruism*). Thus, drawing on the principles of positive psychology, the hope theory and gratitude, and embedding group curative factors, the HOPE program provides participants with a novel toolkit to develop skills and resources to improve their well-being and quality of life during and beyond treatment.

### Objectives

The HOPE program recognizes the common challenges and unmet needs across all types of cancer, including fatigue, fear of recurrence, and psychological distress [6-16]. The HOPE program was co-designed with service users and stakeholders from one of the United Kingdom’s leading cancer charities, Macmillan Cancer Support (MCS) [38,39]. It has been delivered face-to-face to breast cancer survivors and in community settings for all cancer types [38,40]. Initial evaluations demonstrate that cancer survivors participating in the face-to-face program feel more confident and hopeful and valued the peer support element of the program, which made them feel less alone with their problems [38].

MCS experienced difficulties recruiting cancer survivors to the face-to-face program in some regions of the United Kingdom. This mirrors the national and international self-management experience [41]. Digital delivery of interventions can improve access and increase user choice for those who may be unable to physically attend face-to-face programs and those who may prefer a remote, digital intervention [42].

It is unclear if digital interventions can offer the same active intervention ingredients, known as group curative factors [37], that participating in a face-to-face group provides. The growth in social networking potentially offers a strong sense of community, a place for sharing experiences of cancer, and a useful platform for self-management and support [43]. Evidence shows that digital interventions have a positive impact on the quality of life and other health, self-management, and behavioral outcomes in cancer survivors and other long-term conditions [44,45]. Digital interventions also appear to address barriers to participation [46] (eg, physical, psychological, cognitive, economic, social, and cultural factors) in existing face-to-face programs [47]. Therefore, we adapted the existing face-to-face format of the HOPE program to create the digital iHOPE (Help to Overcome Problems Effectively; where the ‘i’ indicates the digital version to distinguish it from the face-to-face HOPE program) program. In this study, we aimed to explore the feasibility of the digital version of the program available for people living with all types of cancer. As a feasibility study, the specific aims were to examine the following aspects:

- Implementation: recruitment rates, completion rates of measures, and adherence rates to the program (ie, number of participants who participated in the sessions)
- Acceptability: satisfaction ratings, ratings of appropriateness and potential usefulness of the program, and indications of positive and negative effects on participants
- Practicality: feedback on the ability to complete activities in terms of time constraints and other personal commitments
- Preliminary efficacy testing: outcomes and effect size estimation [48-50].

## Methods

### Informed Consent

The study was approved by the Coventry University Ethics Committee (P21296). All participants provided informed consent before participation in the study.

### Participants and Recruitment

**Figure 1:**
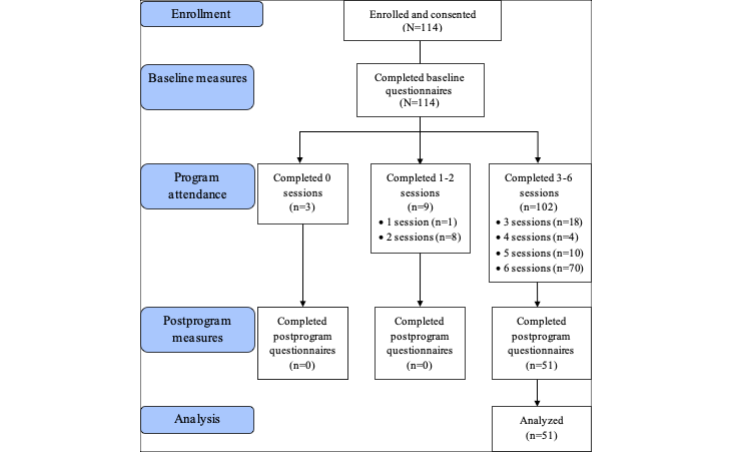
Participant flow.

A convenience sample of cancer survivors recruited to 5 iHOPE programs over 10 months was used (n=114). Eligible study participants were adults (≥18 years), living in the United Kingdom, coming to the end of cancer treatment or surgery, or having recently completed treatment, able to read and understand English, and with access to the internet and an email account. Participants were recruited through advertisement of the program on the MCS Facebook page, Twitter, and Macmillan’s *LearnZone* area on their website. Participants were allocated a place on the program on a first-come, first-served basis. In light of previous digital intervention research showing that participants who completed a minimum of 50% of sessions had a reliable change in outcome measures [51], we categorized participants as having completed the course if they completed three or more of the 6 sessions (n=102). All participants completed the baseline questionnaires (n=114), but only those who also completed the postprogram questionnaires were included in the analysis (n=51). Of the remaining 63 participants who did not complete the postprogram questionnaires, 3 participants did not use the program at all, 9 participants completed 1 or 2 sessions, and 51 participants completed between 3 and 6 sessions (see Figure 1).

### Program Development

Full details of the development of the HOPE program have been published elsewhere [38-40,52,53]. The HOPE program has been taxonomized using the taxonomy of self-management support [54]. The adaptation to the digital iHOPE program was undertaken in consultation with people who had attended, delivered, and commissioned the delivery of the face-to-face HOPE program. A user-centered, iterative approach was undertaken [55], as detailed below. A set of design requirements and a design brief were drawn up in consultation with end users and stakeholders. It was specified that the digital version (iHOPE) should replicate the process and content of the group-based HOPE course to ensure that the marketing and recruitment of both versions of the course would be consistent and that cross-training of face-to-face facilitators to deliver iHOPE would be kept to a minimum. The initial digital version of HOPE went through a number of iterative testing sessions, with improvements made to usability after each iteration. It was intended through these iterations to develop a system that was usable and accepted by the intended user group to increase the likelihood of uptake and continued usage, and ensure the technology did not prove a barrier to engagement and participation.

#### Iteration 1

We conducted usability testing of iHOPE with stakeholders, including MCS staff, trainers, and cancer survivors. A link to the course was circulated to past participants and facilitators of the face-to-face program. They were asked to work through the web-based material and provide feedback to specific questions via email. This study aimed to explore if the core components of HOPE were integrated effectively.

#### Iteration 2 

The iHOPE web-based course was then reviewed by a wider audience and demonstrated to delegates at the National Cancer Voices conference in November 2013. This round of feedback focused on the acceptability of the translation of face-to-face course features into a web format. 

#### Iteration 3

MCS and Coventry University researchers held a workshop with the web design team and experienced HOPE facilitators to collect feedback on iHOPE design, usability, and content. User and facilitator feedback led to further revisions to improve usability and course experience.

#### Iteration 4 

The final iterative feedback was undertaken as part of the iHOPE evaluation, and 5 cohorts reviewed the system while they were enrolled in the program.

### Content

**Figure 2:**
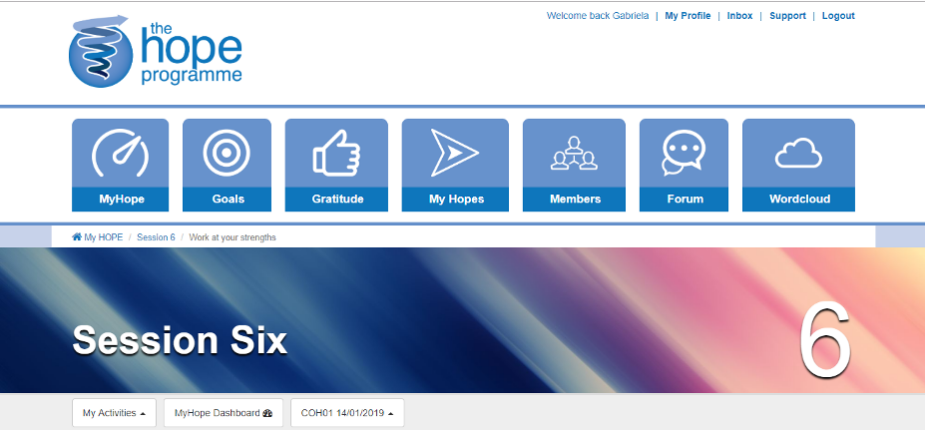
Help to Overcome Problems Effectively user dashboard.

The iHOPE program content comprises text, images, downloadable documents, and links to external websites, for example, activities and media related to cancer-related fatigue and developing character strengths. The content delivered is configured into interactive activities (eg, quizzes, self-monitoring tools, and diaries) that can be used by participants to learn and consolidate program content (Table 1 gives details of iHOPE program content and activities, and Figure 2 shows screenshots of user dashboards). The iHOPE program uses forums and messaging facilities that act as a conduit for communication between participants and facilitators.

**Table 1:** Weekly topics, content, exercises, and activities included in the 6-week Help to Overcome Problems Effectively (where ‘i’ indicates digital version) program.

Session	Examples of content	Examples of exercises and activities (self-management tools)
Week 1: Introduction/instilling hope	Aims of the program User guide to navigating the platform and setting up a profile Introduction to self-management The benefits of positive emotions Video: positive emotions for a flourishing life The power of gratitude Personalized goal setting Video: how to set achievable goals Forum topic: reasons for joining the program Further resources and links (eg, videos, podcasts, and websites) to gratitude, positivity, and goal setting	Interactive gratitude diary SMARTER^a^ goal setting Assessment: positivity ratio test and positive and negative emotions test
Week 2: Stress management	Understanding stress Managing stress Videos: how to manage stress and how to make stress your friend Coping with unhelpful thinking patterns Mindfulness for stress management and meditation Self-compassion and acceptance Video: how to be kind to yourself Forum topic: how do you deal with cancer-related stress? Further resources and links (eg, videos, podcasts, and websites) to self-compassion, mindfulness, and stress management	Interactive gratitude diary SMARTER goal setting and goal feedback Guided relaxation and meditation exercise (podcasts) How to cope with unhelpful thoughts (worksheet)
Week 3: Managing fatigue	Understanding the boom and bust cycle Using the 3 Ps (prioritizing, planning, and pacing) for managing fatigue Video: tips for managing fatigue Sleeping better; podcast: tips to improve sleep Forum topic: coping with fatigue Further resources and links (eg, videos, podcasts, and websites) to sleeping better	Interactive gratitude diary SMARTER goal setting and goal feedback Fatigue and pacing diaries (worksheets) Quiz: What are the main challenges faced by cancer survivors?
Week 4: Body image and communication	Body image Video: body image and cancer Sexuality and intimacy Video: Cancer as a passport to emotional intimacy Communication skills and tips for talking with the health care team and family Forum topic: experiences of coping with body changes and experiences of communicating with the health care team Further resources and links (eg, videos, podcasts, and websites) to sexuality, intimacy, and relationships	Interactive gratitude diary SMARTER goal setting and goal feedback
Week 5: Physical activity and fear of recurrence	Coping with fear of recurrence Videos: Moving forward while being worried about cancer returning and the regrets of those who are dying Hopes and dreams for the future Video: Before I die project The benefits of physical activity Video: Tips for becoming and staying active Forum topic: Concerns about cancer coming back Further resources and links (eg, videos, podcasts, and websites) to managing concerns about cancer coming back and getting more active	Interactive gratitude diary SMARTER goal setting and goal feedback
Week 6: Character strengths and happiness	Understanding how using your strengths can lead to a more fulfilling life Video: The science of character strengths Tips for authentic happiness; managing setbacks and keeping going Forum topic: Learning from the program Further resources and links (eg, videos, podcasts, and websites) to Macmillan Cancer Support online communities and happiness resources	Interactive gratitude diary SMARTER goal setting and goal feedback Assessment: positivity ratio test and positive and negative emotions test and character strengths Quiz: What contributes to happiness?

^a^SMARTER: SMARTER is an acronym used by many organizations for goal setting, and stands for: Specific, Measurable, Achievable, Relevant, Time-bound, Enjoyable, Reward.

**Figure 3:**
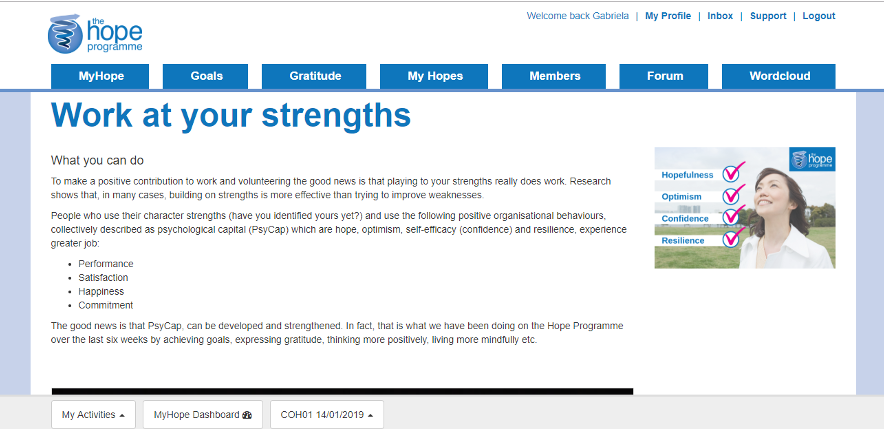
Help to Overcome Problems Effectively personal strengths exercise.

The theory underpinning the HOPE program is described elsewhere [38,53]. Briefly, the iHOPE program aims to enhance well-being by fostering positive emotions and stimulating positive functioning. A parallel goal is to reduce depressive symptoms. The iHOPE program is based on principles derived from positive psychology and focuses on positive experiences, strengths, and personal competencies rather than mental health problems such as anxiety and depression. It incorporates evidence-based exercises based on positive psychology, in addition to elements stemming from mindfulness, cognitive behavioral therapy, and problem-solving therapy (Figure 3 gives an example of personal strength exercises). The use of positive psychology interventions in cancer survivorship has, to date, been limited; however, a systematic review has supported their impact on well-being and quality of life [56].

### Delivery

The maximum group size for each program delivery was set at 20 participants, following consultation with the iHOPE program facilitators and a beta test involving 33 participants. The iHOPE program content is released weekly over 6 weeks, thus mirroring the delivery model of the group-based face-to-face HOPE program. The iHOPE program is an asynchronous program that does not require real-time attendance. On the same day each week, new content is released, whereas previous content remains available. Participants are encouraged to log on for approximately 2.5 hours per week and use a range of behavior change techniques, including weekly goal setting, action planning, and self-monitoring. Peer support and interaction is facilitated through social networking tools and shared interactive activities where all participants’ comments appear. Weekly topics and activities are provided in Table 1.

Goal setting and gratitude activities are recurring weekly features, and participants are encouraged to post a goal or something they may feel grateful for on the *online walls* for everyone to see and comment on. Each week, a number of questions are set as discussion topics that are featured in weekly forums. These questions are related to the program content for each week. There is also a weekly forum (Hope Lounge) where participants can start a discussion about either their experience of living with and beyond cancer or a noncancer related topic.

The iHOPE program is moderated by 2 trained peer facilitators who are affected by cancer in some way. The facilitators received training from MCS and followed a delivery protocol. The facilitator’s role is to offer encouragement to participants, stimulate discussion in social networking forums by inviting participants to respond with comments to specific questions, or respond to questions/comments posted by participants. Facilitators also monitor daily social networking posts for safety and report any technical problems to the research team. Facilitators spent 2 hours each per session, supporting the participants.

### Procedure

One week before each group’s iHOPE program start date, participants were sent a link to a web-based survey to access an introductory letter that explained the purpose of the study, the research participant information sheet, consent form, demographic questionnaire, and outcome measures (ie, baseline questionnaires). Participants completed these and then went on to access the iHOPE program. At the end of the 6-week program, participants were emailed the survey link to complete the outcome measures again (ie, postprogram questionnaires) and complete a usability questionnaire.

### Data Collection

Data collection was designed to address each of the feasibility testing research aims. Demographic data were collected at baseline on participants’ age, sex, ethnicity, marital status, employment status, changes in work hours because of cancer, educational level, and cancer site.

#### Implementation

Recruitment, retention, and completion rates of the measures were recorded. The iHOPE program system captured basic adherence data, in terms of how many participants accessed the program and the number of sessions they accessed. Furthermore, detailed usage data were captured by the number of times participants carried out key program activities, such as goal setting and gratitude diaries.

#### Acceptability and Practicality

We created our own bespoke questionnaire to derive satisfaction scores with specific elements of interest for program development purposes, such as navigation, presentation, the usefulness of specific program elements, and support from the program facilitators. As previous literature shows that the optimal number of potential responses on a scale is between 4 and 7 [45], we presented participants with a choice of 4 responses (ie, strongly disagree, disagree, agree, and strongly agree) in an effort to reduce participant response burden. In addition, as these responses were used to inform program development and changes to future versions, the inclusion of a *neutral* response on the scale would be uninformative. Satisfaction with the program was assessed using a 4-point scale [57] anchored at strongly agree and strongly disagree. The 13 questions (summarized in the Results section and Multimedia Appendix 1) were derived from published research examining the usability of digital self-management interventions [58-60].

#### Outcome Measures for Preliminary Efficacy Testing

All of the following outcome measures were administered at baseline and again at the end of the iHOPE program.

The quality of life in adult cancer survivors scale (QLACS) [61] is a validated questionnaire comprising 47 items across 12 domains, where 7 domains measure the generic quality of life and 5 domains measure the cancer-specific QLACS. In this study, we used 2 subscales of the QLACS that specifically address frequently cited unmet needs for survivors: cancer-related fatigue and cancer-related concern or fear of recurrence. Fatigue is assessed using the following 4 items: *In the past four weeks... (1) you had the energy to do the things you wanted to do [note that this item was reverse-scored]; (2) you felt fatigued; (3) you did not have energy to do the things you wanted to do; and (4) you felt tired a lot*. Fear of cancer recurrence is measured by the following 4 items: *In the past four weeks… (1) you worried about dying from cancer; (2) you worried about cancer coming back; (3) whenever you felt a pain, you worried that it might be cancer again; and (4) you were preoccupied with concerns about cancer*. Each item in these 4-item subscales is scored from 1 to 7 (1=never, 2=seldom, 3=sometimes, 4=about as often as not, 5=frequently, 6=very often, and 7=always). Items are summed to give an overall score of 4 to 28 for each of the 2 subscales (ie, fatigue and fear of recurrence), with higher scores indicating greater difficulties.

The patient health questionnaire-9 [62] is a 9-item measure that assesses the frequency of experience of the symptoms of depression, for example, *Over the past two weeks, how often have you been bothered by any of the following problems... (1) little interest or pleasure in doing things; (2) feeling down, depressed, or hopeless; and (3) poor appetite or overeating*. Responses to each of the 9 items range from 0 to 3 (0=not at all, 1=several days, 2=more than half the days, and 3=nearly every day), leading to a summed score between 0 and 27, with higher scores indicating greater severity of depression. Scores of 10 or more are presumed to be above the clinical range, and so, scores of less than 10 are classified as *cases* of depression. Recovery rates were calculated as those patients who scored ≥10 (cases) before treatment and scored <10 posttreatment.

The generalized anxiety disorder scale [63] is a 7-item scale measuring symptoms of generalized anxiety disorder, for example, *Over the past two weeks, how often have you been bothered by the following problems… (1) feeling nervous, anxious, or on edge; (2) trouble relaxing; and (3) becoming easily annoyed or irritable*. Responses to all 7 items range from 0 to 3 (0=not at all, 1=several days, 2=more than half the days, and 3=nearly every day), providing a total score of 0 to 21, with higher scores indicating greater anxiety. Scores ≥8 are classified as *cases* of generalized anxiety disorder. Recovery rates were calculated for those patients who score ≥8 (cases) before treatment and <8 post-treatment.

The Warwick Edinburgh mental well-being scale [64] is a scale of 14 positively worded feelings and thoughts, used to assess mental well-being within the adult population. The scale includes measures of positive affect, satisfying interpersonal relationships, and positive functioning, for example, *Below are some statements about feelings and thoughts. Please tick the box that best describes your experience of each over the last two weeks… (1) I have been feeling optimistic about the future; (2) I have been thinking clearly; and (3) I have been feeling love*. Participants rated each of the 14 items on a scale of 1 to 5 (1=none of the time, 2=rarely, 3=some of the time, 4=often, and 5=all of the time), providing a total positive mental well-being score ranging from 14 to 70, with higher scores representing greater positive mental well-being. A change of three or more is seen as clinically *meaningful* change [65].

Hope was measured using the 6-item adult state hope scale [66], which assesses goal-directed thinking in any given situation. The scale has 3 agency items, for example, *At the present time, I am energetically pursuing my goals*, and 3 pathway items, for example, *I can think of many ways to reach my current goals*. Participants indicated the extent to which they agree with each of the 6 statements, in accordance with how they feel at the present moment, on a scale of 1 to 8 (1=definitely false, 2=mostly false, 3=somewhat false, 4=slightly false, 5=slightly true, 6=somewhat true, 7=mostly true, and 8=definitely true). Total hope scores range from 6 to 48, with higher scores indicating higher levels of hopeful thinking.

The gratitude questionnaire-6-item form [67] is a self-report measure of disposition to experience gratitude in everyday life. It is a 6-item scale, comprising items such as *(1) I have so much in life to be thankful for, (2) I am grateful to a wide variety of people, and (3) when I look at the world, I do not see much to be grateful for [note that this item is reverse-scored*]. Participants scored each item from 1 to 7 (1=strongly disagree, 2=disagree, 3=slightly disagree, 4=neutral, 5=slightly agree, 6=agree, 7=strongly agree), giving a total score of 6 to 42, where a higher score indicates more gratitude shown [67].

### Analysis

All statistical data analyses were conducted using IBM SPSS Statistics 25 (IBM Corp Released 2017. IBM SPSS Statistics for Windows, Version 25.0). The sample was not powered to detect significance in the outcome measures; nevertheless, we present changes in the scores to aid understanding of the potential effect of the program and to provide data on which to base a power calculation for a larger study of efficacy. The level of statistical significance was set at *P*<.05. Owing to some deviations from the normal distribution in follow-up gratitude, anxiety, and depression scores, all variables were analyzed using Wilcoxon signed-ranks for paired samples. We report the means with SDs and medians with a range of scores. As recommended for nonparametric paired data, we report *r* as the effect size estimates (r=z/√n), where z is calculated through the Wilcoxon test and n is the number of observations (n=102) [68]. Recommended boundaries for *r* were used to determine small (0.1), moderate (0.3), and large effect sizes (0.5) [69].

## Results

### Descriptive Data

A total of 114 participants completed the baseline questionnaires, and those who also completed the postprogram questionnaires were included in the analysis (n=51). Participant characteristics are shown in Table 2. Participants were predominately female, white ethnicity, married, with educational qualifications, and employed. The majority had cut work hours because of their cancer, and 38.6% (44/114) had breast cancer; however, data on cancer site was incomplete as participants did not always share this. There were no significant differences between these subgroups of participants on any of the demographic variables.

Examining baseline depression, anxiety, well-being, quality of life, cancer-related fatigue and worry, hope, and gratitude scores between those who did and did not complete postprogram questionnaires revealed no significant differences (scores are summarized in Multimedia Appendix 2).

**Table 2:** Participant demographic information at enrolment, baseline, and postprogram.

Characteristics		Sample at enrolment (N=114)	Completed baseline questionnaire only (n=63)	Completed baseline and postprogram questionnaire (n=51)
Age, mean (SD)		51.3 (9.6)^a^	47.4 (8.8)^b^	53.6 (9.4)^c^
Female, n (%)		102 (89.5)	56 (89)	46 (90)
White ethnicity, n (%)		111 (97.4)	61 (97)	50 (98)
Married or living with partner, n (%)		86 (75.4)	46 (73)	40 (78)
Employed, n (%)		68 (59.6)	36 (57)	32 (63)
Cut work hours because of cancer, n (%)		73 (64.0)	43 (68)	30 (59)
Possessed postschool qualifications, n (%)		110 (96.5)	60 (95)	50 (98)
**Cancer type, n (%)**				
	Breast	44 (38.6)	22 (35)	22 (43)
	Gynecological	13 (11.4)	6 (10)	7 (14)
	Other	36 (31.6)	21 (33)	15 (29)
	Not reported	21 (18.4)	14 (22)	7 (14)

^a^n=75 because of missing data.

^b^n=27 because of missing data.

^c^n=48 because of missing data.

### Implementation

A total of 114 people were recruited to the program, of which 102 participants completed 3 or more sessions (89.4% completion of the program). The mean number of sessions was 5.0 (SD 1.5), and 61.4% (70/114) of participants attended all 6 sessions. Half of those who completed 3 or more sessions of the program (n=51) went on to complete the outcome measures, giving a 50% response rate to questionnaires at the end of the program.

### Acceptability and Practicality

The 59 participant responses to the satisfaction questionnaire are detailed in Multimedia Appendix 2. The overwhelming majority of participants *strongly agreed* or *agreed* that the program and its delivery were positive and activities in the program were useful.

### Preliminary Efficacy Testing

Table 3 provides a summary of all outcome measures for the 51 participants who completed baseline and postprogram questionnaires.

**Table 3:** Baseline and postprogram outcome scores (n=51).

Outcome measure^a,b^	Baseline scores, mean (SD)	Baseline scores, median (range)	Postprogram scores, mean (SD)	Postprogram scores, median (range)	*P *value^c^	*r *value^d^
Depression	8.9 (5.2)	9 (0-20)	6.1 (4.9)	5 (0-25)	<.001	−0.46
Anxiety	6.8 (4.9)	5 (0-20)	4.1 (4.1)	3 (0-18)	<.001	−0.35
Positive mental well-being	42.2 (9.3)	43 (21-63)	49.9 (7.5)	51 (25-67)	<.001	−0.50
Fatigue	18.1 (5.7)	19 (5-28)	14.4 (6.4)	13 (4-27)	<.001	−0.44
Fear of recurrence	15.9 (6.3)	13 (8-28)	13.2 (6.0)	12 (4-28)	<.001	−0.37
Hope	28.0 (10.0)	28 (8-47)	35.3 (7.4)	36 (11-46)	<.001	−0.51
Gratitude	34.8 (5.3)	36 (16-42)	36.7 (5.1)	38 (14-42)	.02	−0.24

^a^For depression, anxiety, fatigue, and fear of recurrence measures, decreasing scores indicate improvement.

^b^For positive mental well-being, hope, and gratitude measures, increasing scores indicate improvement.

^c^*P* value based on Wilcoxon signed-ranks.

^d^Effect size of change.

### Psychological Distress

At the end of the iHOPE program, participants reported statistically significant improvements in depression and anxiety, with moderate effect sizes. Before the program, 47% (24/51) of participants exceeded the clinical cutoff scores for depression, and 43% (22/51) of participants exceeded the clinical cutoff scores for anxiety. At the end of the program, 31% (16/51) and 29% (15/51) of participants had scores that indicated a *recovery* from depression (*z*=−4.607; *P*<.001; *r*=−0.46) and anxiety (*z*=−3.534; *P*<.001; *r*=−0.35), respectively (ie, their scores were no longer above the threshold for clinical levels of distress).

### Quality of Life for Cancer-Related Concerns

At the end of the iHOPE program, participants reported statistically significant improvements in cancer-related fatigue (*z*=−4.421; *P*<.001; *r*=−0.44) and *worry* or fear of recurrence (*z*=−3.765; *P*<.001; *r*=−0.37), with moderate effect sizes.

### Positive Well-Being, Gratitude, and Hope

At the end of the iHOPE program, participants reported statistically significant improvements in positive mental well-being (*z*=−5.075; *P*<.001; *r*=−0.50) and hope (*z*=−5.113; *P*<.001; *r*=−0.51), with a moderate effect size for well-being and a large effect size for hope. Participants also reported statistically significant improvements in gratitude (*z*=−2.422; *P*=.015; *r*=−0.24), but with a small effect size (Table 3).

## Discussion

### Principal Findings

This study aimed to describe the development, evaluation, and feasibility of a digital self-management program for people who are living with, or have survived, all types of cancer. The program was well attended, with 114 participants at enrolment and 102 participants (89.5%) completing at least three of the six sessions. Feedback from the participants who completed the satisfaction questionnaire was overwhelmingly positive for many aspects of the content and design. Preliminary efficacy testing had positive results, with generally moderate-sized effects in the expected direction.

The majority of the participants were female, white, married, employed, and educated. Although this may present a limitation in terms of generalizing the study results to other demographic groups (eg, genders, cancer sites, ethnicities, and socioeconomic groups), a recent systematic review has reported that the majority of participants in self-management cancer programs are indeed women [26], and so, this study is representative in this respect. A low attendance rate for men is common in self-management and is linked to their reluctance to seek help [70]. Men are more likely to respond to marketing and recruitment messages that emphasize stoicism, independence, and control [70] and where marketing and recruitment materials contain images of men [71]. Our research group has begun looking at the self-management priorities of men diagnosed with cancer and the types of digital support they prefer [72,73]. It will be vital to conduct further development work to explore how to reach males, members of other ethnic groups, unemployed, and people with lower educational attainment. Ensuring that recruitment materials contain images and messages that appeal to multiple audiences and that recruitment and advertising take place in areas and locations frequented by people of all ages, ethnicities, genders, and income groups has been shown to widen participation in other studies [71]. These recruitment strategies will be suggested to MCS for future recruitment to the digital iHOPE program, as recommendations to reduce inequalities in care provision and recruitment bias.

Reporting rates of cancer sites were suboptimal, and data collection procedures should be improved, perhaps with a user-friendly checklist of cancer sites. Furthermore, collection of data on time since diagnosis and/or stage of treatment will allow us to verify in future studies if those recently diagnosed with cancer will show the same postprogram improvements in outcome measures as those who have survived cancer (or are coming to the end of treatment). Of those providing data, the largest group was breast cancer, reflecting national data. Gynecological cancers were reported by 11.4% of participants, which is greater than the estimated 5% of the population incidence [74]. However, the iHOPE program recognizes commonalities across types of cancer in the challenges faced and in unmet psychosocial needs; therefore, the majority of the content is relevant to all cancer survivors regardless of the type of cancer. This pragmatic approach is a particular strength of the iHOPE program, especially as some patients may have cancer in more than one primary cancer site. Nevertheless, there are some elements of the iHOPE program content that may be more or less applicable to certain groups. In future cohorts, the iHOPE program could be offered to patients at all stages of treatment and/or with the same type of cancer. Another option is to adapt the program for some of the most common types of cancers, so that depending on the users’ profile, personalized content is delivered. For example, in the body image session, prostate cancer and breast cancer users are provided with information, case studies, and other materials specific to their needs. Research has shown that personalized content improves engagement and retention.

The implementation of the program had positive results—89% of the participants completed the program (ie, attended 3 or more sessions) [51], and the average number of sessions attended was 5. Overall, 61% completed all 6 sessions, which is in line with rates of full program completion in other digital interventions, where a median of 56% of participants completed the full program [51,75].

There is a distinction between *dropout attrition* where participants do not fill in questionnaires (ie, 50% in this study) and *nonusage attrition* where participants stopped using the program (ie, only 11% completed <3 sessions in this study) [76]. High rates of nonusage attrition [76], are common and of concern in digitally delivered interventions. Reporting and analyzing the data at the level of session completion is crude, and a more useful and nuanced understanding of engagement and attrition is being developed, but still in its infancy. Researchers are beginning to use a variety of tools such as visualization and log data analysis, which provide evidence of features and content usage over time and detect usability issues. This type of analysis has the potential to improve user experience and improve engagement and outcomes. It is worth noting that some studies have not shown a linear relationship between time spent, the number of sessions completed, and outcomes [51]. It is possible that the *nonusage* participants in this study may be *e-attainers* (as described in [77]), where these participants may have left the program before completion but achieved what they needed from the program, such as learning about goal setting and stress management or obtaining reassurance and relief that their challenges and concerns are shared by others [37]. A higher rate of noncompletion of postprogram measures was observed in this digital delivery of iHOPE (ie, 50%) than the response rate in a face-to-face trial of the cancer HOPE program (88%, unpublished data). We suggest that the high noncompletion rate in this study was partly because of the timing of the research—data collection occurred around the Christmas period. Avoiding holiday times [78], using behavioral prompts [79], and reducing the number of outcome measures to reduce respondent burden [80] could improve data completion rates in future studies assessing the feasibility and practicality of digital cancer self-management programs.

The program was acceptable and appears to be practical, with positive feedback on program activities. The program uses peer support features and is supported by a facilitator with personal experience of cancer. There is a growing evidence base showing that trained peers can respond safely and therapeutically to distressing issues that often arise during self-management programs [81]. This study suggests the potential for the web environment to also provide peer support benefits to cancer survivors. The outcomes we measured capture important challenges for cancer survivors. Several activities in the iHOPE program directly address fatigue and worry, with a fatigue management session comprising video material and pacing and fatigue diaries, in addition to sessions on relaxation and mindfulness [82].

Acknowledging the significant limitations owing to the 50% completion rate of postprogram questionnaires, the findings show that scores for depression, anxiety, well-being, cancer-related fatigue and worry, hope, and gratitude were improved postprogram for those who completed the questionnaires. Moderate effect size improvements were achieved for most of the outcomes, which is consistent with other self-management research for long-term conditions involving self-selecting participants [83]. The fact that some of these improvements were evident in such a short period (6 weeks for postprogram outcomes) is extremely encouraging and consistent with early improvements found in brief self-management programs [84]. The variables likely to be used as clinical outcome variables (depression, anxiety, well-being, fatigue, and worry) all achieved moderate effect sizes in our uncontrolled pre-post study. Considering a future randomized controlled trial and taking into account the limitations of this study, a generic sample size calculation for moderate effect size (power 0.95, comparing 2 groups over 2 time points) would lead to a required total sample size of 132 (66 in each arm) [85].

The need for better care for comorbid depression in cancer survivors has been called for [14,19]. Many participants exceeded clinical cutoff values for depression (47%) and anxiety (43%) when they started the program. This indicates a high rate of clinical depression and anxiety among this population, therefore supporting the need for, and provision of, programs such as iHOPE. Further resources are needed to provide a range of interventions across the NHS for people with depression and anxiety following cancer. These resources could include a digital self-management program as part of a comprehensive stepped care package, particularly as the provision of expert mental health care across Europe remains limited [86].

By the end of the iHOPE program, the scores indicated that nearly one-third (16/51, 31%) had *recovered* from depression and over a quarter (15/51, 29%) had *recovered* from anxiety. These results are encouraging, compare well with general data on short-term psychological interventions [87], and suggest that further research investigating the effectiveness of the iHOPE program is imperative. The improved scores in gratitude and hope are reassuring, as this program is based on these core positive psychology concepts. Gratitude is linked to fewer health complaints, feeling more attentive, more energetic, more determined, more satisfied with life, more optimistic, having more feelings of connection to others, and being more likely to give and use social support [88]. The iHOPE program aims to increase hope among participants by encouraging participants to set and achieve weekly goals. Weekly goal setting and feedback are important factors in initiating behavior change [89]. To date, there are very few hope-based, goal setting interventions that have been evaluated among cancer survivors [56], supporting the need for further development of the iHOPE program. An important next step is to formally explore if changes in hope and gratitude are important mechanisms of change for depression, anxiety, quality of life, and positive mental well-being.

### Limitations

Our findings from this study are limited by the lack of a control group; lack of longer-term follow-up; small sample size; and a sample mainly of white, married women with a high level of education. Participants were recruited via the MCS charity website, Facebook, and other social media sites. This is likely to have attracted highly motivated cancer survivors, with good health and digital literacy skills, compared with cancer survivors who do not access health websites. Further studies of the feasibility and acceptability of the iHOPE program could utilize recruitment strategies aimed at widening participation for male cancer survivors [72,73] and participants from ethnic minorities and low-income backgrounds [71]. Further research is required to understand what drives some participants toward a digital intervention and to work with those with cancers in other sites to ensure that there are no barriers to accessing the iHOPE program.

There was a low response to the postprogram questionnaire. It is possible that there is a bias in responding to those who did find the program useful. The results of this study should be considered with caution, particularly in relation to the generalization of data. However, this feasibility testing suggests there is acceptability and practicality to our program, but there is a further requirement to support retention to complete research measures in future programs. This study used a crude usage attrition assessment. Future studies would benefit from using more sophisticated tracking tools and data analysis to reduce usage attrition and improve engagement and outcomes. Future research should now be a randomized controlled trial, powered to detect significant changes, a longer follow-up, and continued measurement of health and well-being related outcomes, satisfaction, and program usage.

### Conclusions

We have shown that a digital self-management program, iHOPE, has the potential to improve several common and pressing unmet needs of cancer survivors, including fatigue and worry, depression, and anxiety. These improvements need to be further tested using a more robust research design involving a much larger sample over a longer time frame within a randomized controlled trial.

## Appendix

Multimedia Appendix 1 Participant responses to the satisfaction and usability questionnaire.

Multimedia Appendix 2 Baseline scores for outcome variables between those who did (n=51) and did not (n=63) complete post-programme questionnaires.

## Abbreviations

HOPE: Help to Overcome Problems Effectively
iHOPE: Help to Overcome Problems Effectively (where ‘i’ indicates digital version)
MCS: Macmillan Cancer Support
NHS: National Health Service
QLACS: Quality of Life in Adult Cancer Survivors scale
