# Supplementary figures and images for: Help to Overcome Problems Effectively for Cancer Survivors: Development and Evaluation of a Digital Self-Management Program
Source: J Med Internet Res. 2020 May 19;22(5):e17824. doi: 10.2196/17824 (PMC7268001; doi:10.2196/17824)

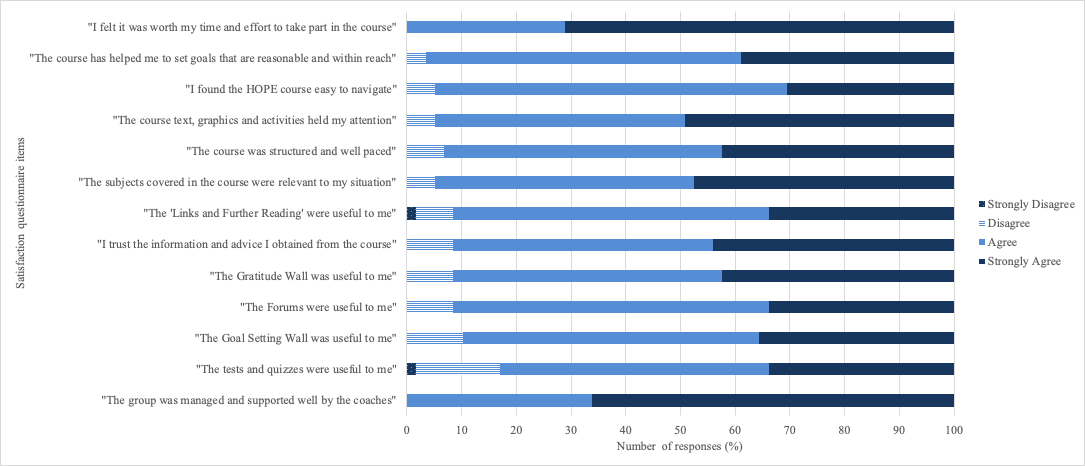

Supplement: Multimedia Appendix 1 [file jmir_v22i5e17824_app1.png]

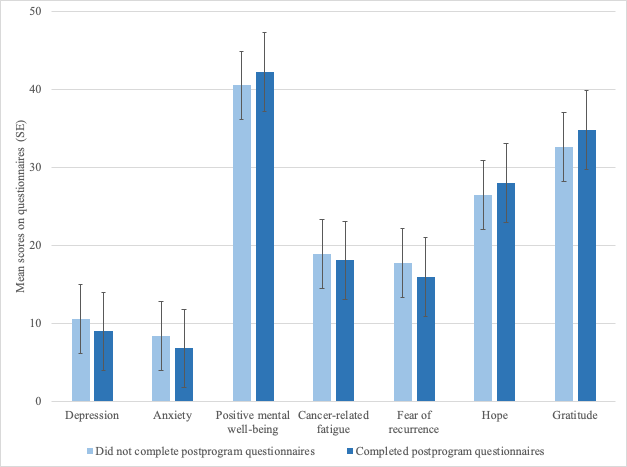

Supplement: Multimedia Appendix 2 [file jmir_v22i5e17824_app2.png]
